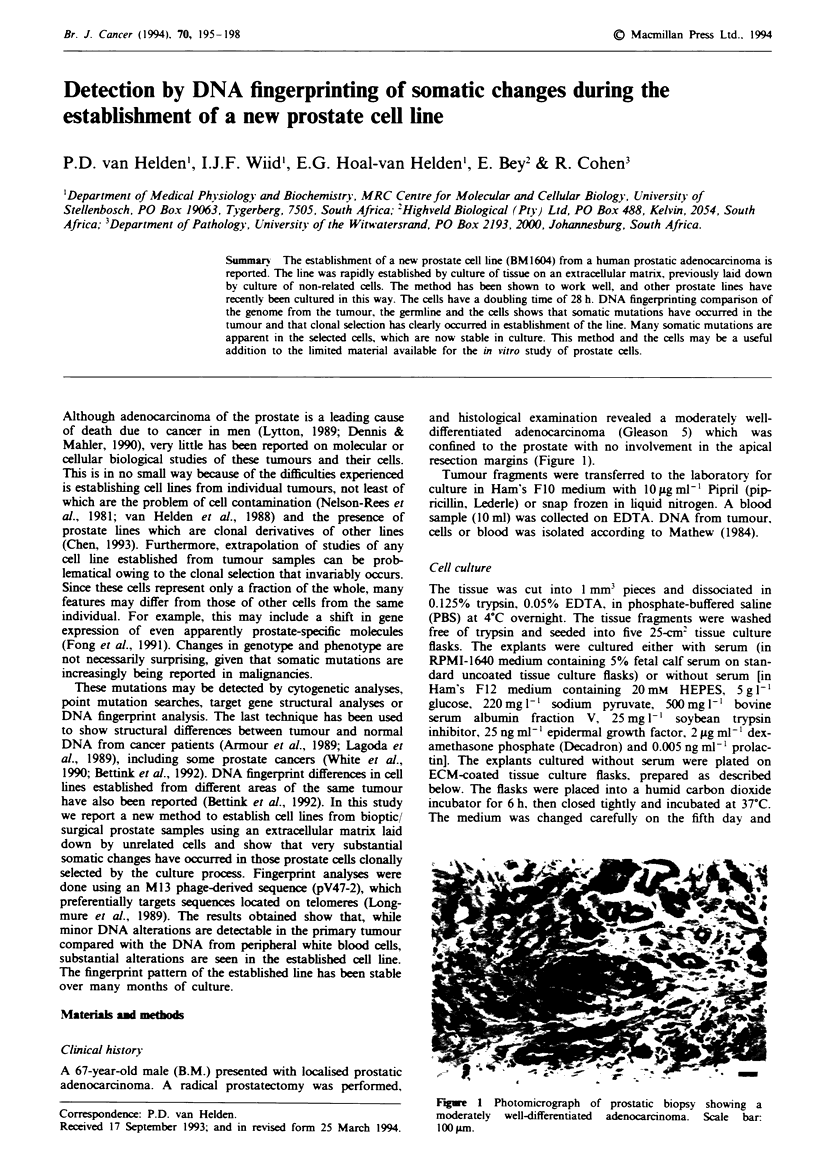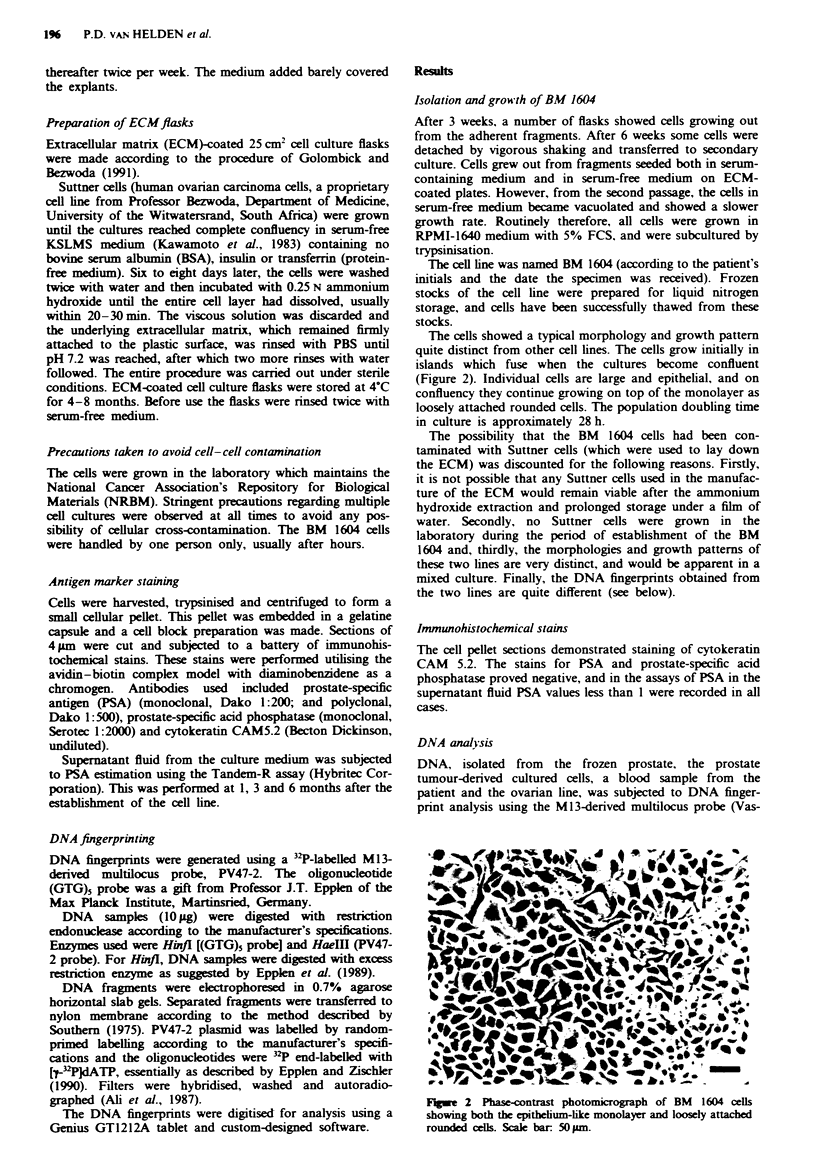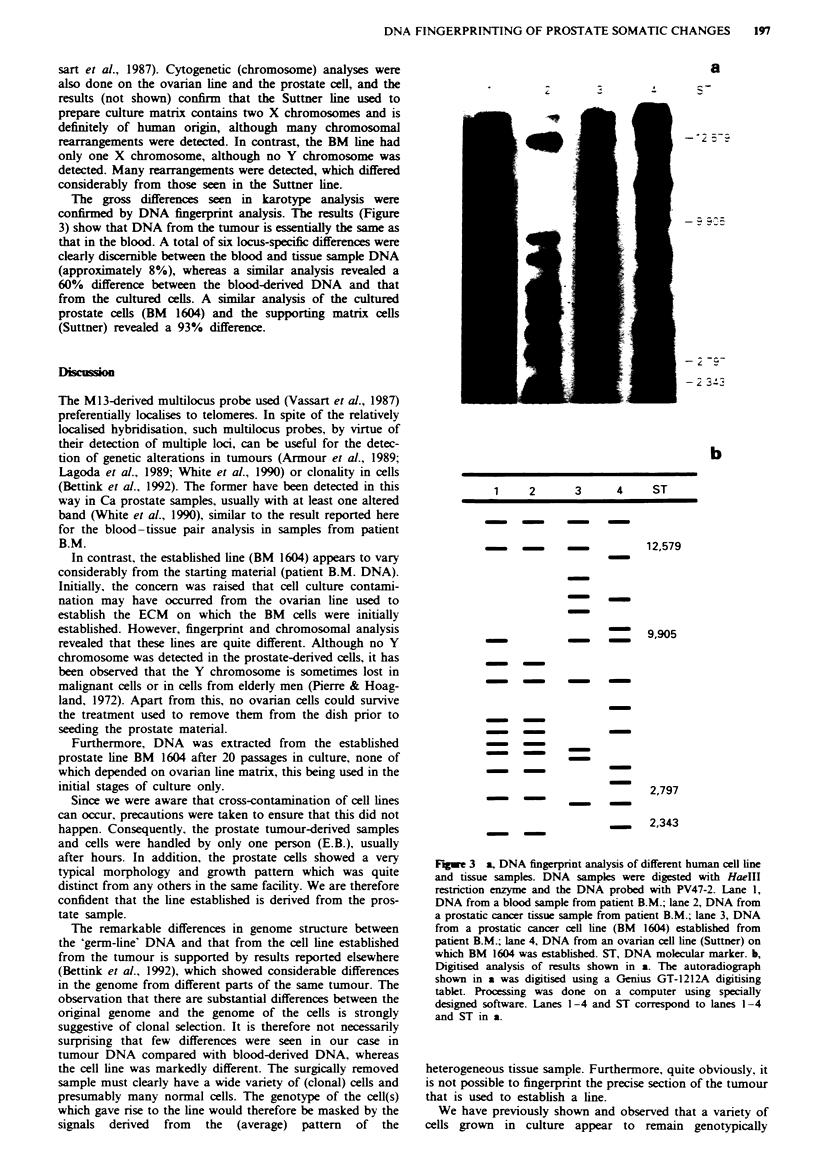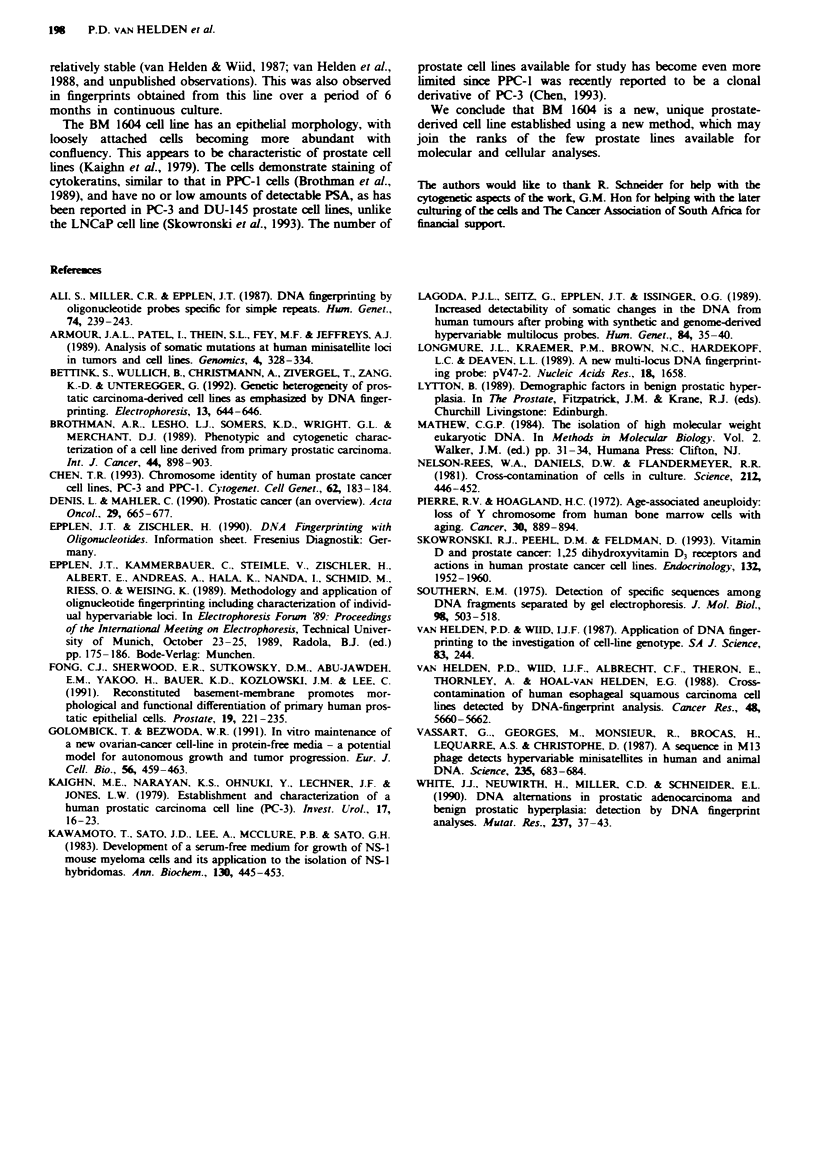# Detection by DNA fingerprinting of somatic changes during the establishment of a new prostate cell line.

**DOI:** 10.1038/bjc.1994.279

**Published:** 1994-08

**Authors:** P. D. van Helden, I. J. Wiid, E. G. Hoal-van Helden, E. Bey, R. Cohen

**Affiliations:** Department of Medical Physiology and Biochemistry, University of Stellenbosch, Tygerberg, South Africa.

## Abstract

**Images:**


Br. J. Cancer (1994), 70, 195-198 © Macmillan Press Ltd., 1994

### Detection by DNA fingerprinting of somatic changes during the establishment of a new prostate cell line

P.D. van Helden[1], I.J.F. Wiid[1], E.G. Hoal-van Helden[1], E. Bey[2] & R. Cohen[3]

[1]*Department of Medical Physiology and Biochemistry, MRC Centre for Molecular and Cellular Biology, University of Stellenbosch, PO Box 19063, Tygerberg, 7505, South Africa;* [2]*Highveld Biological (Pty) Ltd, PO Box 488, Kelvin, 2054, South Africa;* [3]*Department of Pathology, University of the Witwatersrand, PO Box 2193, 2000, Johannesburg, South Africa.*

**Summary** The establishment of a new prostate cell line (BM1604) from a human prostatic adenocarcinoma is reported. The line was rapidly established by culture of tissue on an extracellular matrix, previously laid down by culture of non-related cells. The method has been shown to work well, and other prostate lines have recently been cultured in this way. The cells have a doubling time of 28 h. DNA fingerprinting comparison of the genome from the tumour, the germline and the cells shows that somatic mutations have occurred in the tumour and that clonal selection has clearly occurred in establishment of the line. Many somatic mutations are apparent in the selected cells, which are now stable in culture. This method and the cells may be a useful addition to the limited material available for the *in vitro* study of prostate cells.

Although adenocarcinoma of the prostate is a leading cause of death due to cancer in men (Lytton, 1989; Dennis & Mahler, 1990), very little has been reported on molecular or cellular biological studies of these tumours and their cells. This is in no small way because of the difficulties experienced is establishing cell lines from individual tumours, not least of which are the problem of cell contamination (Nelson-Rees *et al.*, 1981; van Helden *et al.*, 1988) and the presence of prostate lines which are clonal derivatives of other lines (Chen, 1993). Furthermore, extrapolation of studies of any cell line established from tumour samples can be problematical owing to the clonal selection that invariably occurs. Since these cells represent only a fraction of the whole, many features may differ from those of other cells from the same individual. For example, this may include a shift in gene expression of even apparently prostate-specific molecules (Fong *et al.*, 1991). Changes in genotype and phenotype are not necessarily surprising, given that somatic mutations are increasingly being reported in malignancies.

These mutations may be detected by cytogenetic analyses, point mutation searches, target gene structural analyses or DNA fingerprint analysis. The last technique has been used to show structural differences between tumour and normal DNA from cancer patients (Armour *et al.*, 1989; Lagoda *et al.*, 1989), including some prostate cancers (White *et al.*, 1990; Bettink *et al.*, 1992). DNA fingerprint differences in cell lines established from different areas of the same tumour have also been reported (Bettink *et al.*, 1992). In this study we report a new method to establish cell lines from bioptic/surgical prostate samples using an extracellular matrix laid down by unrelated cells and show that very substantial somatic changes have occurred in those prostate cells clonally selected by the culture process. Fingerprint analyses were done using an M13 phage-derived sequence (pV47-2), which preferentially targets sequences located on telomeres (Longmure *et al.*, 1989). The results obtained show that, while minor DNA alterations are detectable in the primary tumour compared with the DNA from peripheral white blood cells, substantial alterations are seen in the established cell line. The fingerprint pattern of the established line has been stable over many months of culture.

### Materials and methods

### *Clinical history*

A 67-year-old male (B.M.) presented with localised prostatic adenocarcinoma. A radical prostatectomy was performed, and histological examination revealed a moderately well-differentiated adenocarcinoma (Gleason 5) which was confined to the prostate with no involvement in the apical resection margins (Figure 1).

Tumour fragments were transferred to the laboratory for culture in Ham's F10 medium with 10 μg $ml^{-1}$ Pipril (pipricillin, Lederle) or snap frozen in liquid nitrogen. A blood sample (10 ml) was collected on EDTA. DNA from tumour, cells or blood was isolated according to Mathew (1984).

### *Cell culture*

The tissue was cut into 1 $mm^3$ pieces and dissociated in 0.125% trypsin, 0.05% EDTA, in phosphate-buffered saline (PBS) at 4°C overnight. The tissue fragments were washed free of trypsin and seeded into five 25-$cm^2$ tissue culture flasks. The explants were cultured either with serum (in RPMI-1640 medium containing 5% fetal calf serum on standard uncoated tissue culture flasks) or without serum [in Ham's F12 medium containing 20 mM HEPES, 5 g $l^{-1}$ glucose, 220 mg $l^{-1}$ sodium pyruvate, 500 mg $l^{-1}$ bovine serum albumin fraction V, 25 mg $l^{-1}$ soybean trypsin inhibitor, 25 ng $ml^{-1}$ epidermal growth factor, 2 μg $ml^{-1}$ dexamethasone phosphate (Decadron) and 0.005 ng $ml^{-1}$ prolactin]. The explants cultured without serum were plated on ECM-coated tissue culture flasks, prepared as described below. The flasks were placed into a humid carbon dioxide incubator for 6 h, then closed tightly and incubated at 37°C. The medium was changed carefully on the fifth day and

**Figure 1** Photomicrograph of prostatic biopsy showing a moderately well-differentiated adenocarcinoma. Scale bar: 100 μm.

Correspondence: P.D. van Helden.
Received 17 September 1993; and in revised form 25 March 1994.

thereafter twice per week. The medium added barely covered the explants.

*Preparation of ECM flasks*

Extracellular matrix (ECM)-coated 25 $cm^2$ cell culture flasks were made according to the procedure of Golombick and Bezwoda (1991).

Suttner cells (human ovarian carcinoma cells, a proprietary cell line from Professor Bezwoda, Department of Medicine, University of the Witwatersrand, South Africa) were grown until the cultures reached complete confluency in serum-free KSLMS medium (Kawamoto *et al.*, 1983) containing no bovine serum albumin (BSA), insulin or transferrin (protein-free medium). Six to eight days later, the cells were washed twice with water and then incubated with 0.25 N ammonium hydroxide until the entire cell layer had dissolved, usually within 20–30 min. The viscous solution was discarded and the underlying extracellular matrix, which remained firmly attached to the plastic surface, was rinsed with PBS until pH 7.2 was reached, after which two more rinses with water followed. The entire procedure was carried out under sterile conditions. ECM-coated cell culture flasks were stored at 4°C for 4–8 months. Before use the flasks were rinsed twice with serum-free medium.

*Precautions taken to avoid cell–cell contamination*

The cells were grown in the laboratory which maintains the National Cancer Association's Repository for Biological Materials (NRBM). Stringent precautions regarding multiple cell cultures were observed at all times to avoid any possibility of cellular cross-contamination. The BM 1604 cells were handled by one person only, usually after hours.

*Antigen marker staining*

Cells were harvested, trypsinised and centrifuged to form a small cellular pellet. This pellet was embedded in a gelatine capsule and a cell block preparation was made. Sections of 4 µm were cut and subjected to a battery of immunohistochemical stains. These stains were performed utilising the avidin–biotin complex model with diaminobenzidene as a chromogen. Antibodies used included prostate-specific antigen (PSA) (monoclonal, Dako 1:200; and polyclonal, Dako 1:500), prostate-specific acid phosphatase (monoclonal, Serotec 1:2000) and cytokeratin CAM5.2 (Becton Dickinson, undiluted).

Supernatant fluid from the culture medium was subjected to PSA estimation using the Tandem-R assay (Hybritec Corporation). This was performed at 1, 3 and 6 months after the establishment of the cell line.

*DNA fingerprinting*

DNA fingerprints were generated using a $^{32}P$-labelled M13-derived multilocus probe, PV47-2. The oligonucleotide $(GTG)_5$ probe was a gift from Professor J.T. Epplen of the Max Planck Institute, Martinsried, Germany.

DNA samples (10 µg) were digested with restriction endonuclease according to the manufacturer's specifications. Enzymes used were *Hinf*I [$(GTG)_5$ probe] and *Hae*III (PV47-2 probe). For *Hinf*I, DNA samples were digested with excess restriction enzyme as suggested by Epplen *et al.* (1989).

DNA fragments were electrophoresed in 0.7% agarose horizontal slab gels. Separated fragments were transferred to nylon membrane according to the method described by Southern (1975). PV47-2 plasmid was labelled by random-primed labelling according to the manufacturer's specifications and the oligonucleotides were $^{32}P$ end-labelled with [$\gamma$-$^{32}P$]dATP, essentially as described by Epplen and Zischler (1990). Filters were hybridised, washed and autoradiographed (Ali *et al.*, 1987).

The DNA fingerprints were digitised for analysis using a Genius GT1212A tablet and custom-designed software.

**Results**

*Isolation and growth of BM 1604*

After 3 weeks, a number of flasks showed cells growing out from the adherent fragments. After 6 weeks some cells were detached by vigorous shaking and transferred to secondary culture. Cells grew out from fragments seeded both in serum-containing medium and in serum-free medium on ECM-coated plates. However, from the second passage, the cells in serum-free medium became vacuolated and showed a slower growth rate. Routinely therefore, all cells were grown in RPMI-1640 medium with 5% FCS, and were subcultured by trypsinisation.

The cell line was named BM 1604 (according to the patient's initials and the date the specimen was received). Frozen stocks of the cell line were prepared for liquid nitrogen storage, and cells have been successfully thawed from these stocks.

The cells showed a typical morphology and growth pattern quite distinct from other cell lines. The cells grow initially in islands which fuse when the cultures become confluent (Figure 2). Individual cells are large and epithelial, and on confluency they continue growing on top of the monolayer as loosely attached rounded cells. The population doubling time in culture is approximately 28 h.

The possibility that the BM 1604 cells had been contaminated with Suttner cells (which were used to lay down the ECM) was discounted for the following reasons. Firstly, it is not possible that any Suttner cells used in the manufacture of the ECM would remain viable after the ammonium hydroxide extraction and prolonged storage under a film of water. Secondly, no Suttner cells were grown in the laboratory during the period of establishment of the BM 1604 and, thirdly, the morphologies and growth patterns of these two lines are very distinct, and would be apparent in a mixed culture. Finally, the DNA fingerprints obtained from the two lines are quite different (see below).

*Immunohistochemical stains*

The cell pellet sections demonstrated staining of cytokeratin CAM 5.2. The stains for PSA and prostate-specific acid phosphatase proved negative, and in the assays of PSA in the supernatant fluid PSA values less than 1 were recorded in all cases.

*DNA analysis*

DNA, isolated from the frozen prostate, the prostate tumour-derived cultured cells, a blood sample from the patient and the ovarian line, was subjected to DNA fingerprint analysis using the M13-derived multilocus probe (Vas-

**Figure 2** Phase-contrast photomicrograph of BM 1604 cells showing both the epithelium-like monolayer and loosely attached rounded cells. Scale bar: 50 µm.

sart *et al.*, 1987). Cytogenetic (chromosome) analyses were also done on the ovarian line and the prostate cell, and the results (not shown) confirm that the Suttner line used to prepare culture matrix contains two X chromosomes and is definitely of human origin, although many chromosomal rearrangements were detected. In contrast, the BM line had only one X chromosome, although no Y chromosome was detected. Many rearrangements were detected, which differed considerably from those seen in the Suttner line.

The gross differences seen in karotype analysis were confirmed by DNA fingerprint analysis. The results (Figure 3) show that DNA from the tumour is essentially the same as that in the blood. A total of six locus-specific differences were clearly discernible between the blood and tissue sample DNA (approximately 8%), whereas a similar analysis revealed a 60% difference between the blood-derived DNA and that from the cultured cells. A similar analysis of the cultured prostate cells (BM 1604) and the supporting matrix cells (Suttner) revealed a 93% difference.

### Discussion

The M13-derived multilocus probe used (Vassart *et al.*, 1987) preferentially localises to telomeres. In spite of the relatively localised hybridisation, such multilocus probes, by virtue of their detection of multiple loci, can be useful for the detection of genetic alterations in tumours (Armour *et al.*, 1989; Lagoda *et al.*, 1989; White *et al.*, 1990) or clonality in cells (Bettink *et al.*, 1992). The former have been detected in this way in Ca prostate samples, usually with at least one altered band (White *et al.*, 1990), similar to the result reported here for the blood–tissue pair analysis in samples from patient B.M.

In contrast, the established line (BM 1604) appears to vary considerably from the starting material (patient B.M. DNA). Initially, the concern was raised that cell culture contamination may have occurred from the ovarian line used to establish the ECM on which the BM cells were initially established. However, fingerprint and chromosomal analysis revealed that these lines are quite different. Although no Y chromosome was detected in the prostate-derived cells, it has been observed that the Y chromosome is sometimes lost in malignant cells or in cells from elderly men (Pierre & Hoagland, 1972). Apart from this, no ovarian cells could survive the treatment used to remove them from the dish prior to seeding the prostate material.

Furthermore, DNA was extracted from the established prostate line BM 1604 after 20 passages in culture, none of which depended on ovarian line matrix, this being used in the initial stages of culture only.

Since we were aware that cross-contamination of cell lines can occur, precautions were taken to ensure that this did not happen. Consequently, the prostate tumour-derived samples and cells were handled by only one person (E.B.), usually after hours. In addition, the prostate cells showed a very typical morphology and growth pattern which was quite distinct from any others in the same facility. We are therefore confident that the line established is derived from the prostate sample.

The remarkable differences in genome structure between the 'germ-line' DNA and that from the cell line established from the tumour is supported by results reported elsewhere (Bettink *et al.*, 1992), which showed considerable differences in the genome from different parts of the same tumour. The observation that there are substantial differences between the original genome and the genome of the cells is strongly suggestive of clonal selection. It is therefore not necessarily surprising that few differences were seen in our case in tumour DNA compared with blood-derived DNA, whereas the cell line was markedly different. The surgically removed sample must clearly have a wide variety of (clonal) cells and presumably many normal cells. The genotype of the cell(s) which gave rise to the line would therefore be masked by the signals derived from the (average) pattern of the heterogeneous tissue sample. Furthermore, quite obviously, it is not possible to fingerprint the precise section of the tumour that is used to establish a line.

**Figure 3** **a**, DNA fingerprint analysis of different human cell line and tissue samples. DNA samples were digested with *Hae*III restriction enzyme and the DNA probed with PV47-2. Lane 1, DNA from a blood sample from patient B.M.; lane 2, DNA from a prostatic cancer tissue sample from patient B.M.; lane 3, DNA from a prostatic cancer cell line (BM 1604) established from patient B.M.; lane 4, DNA from an ovarian cell line (Suttner) on which BM 1604 was established. ST, DNA molecular marker. **b**, Digitised analysis of results shown in **a**. The autoradiograph shown in **a** was digitised using a Genius GT-1212A digitising tablet. Processing was done on a computer using specially designed software. Lanes 1–4 and ST correspond to lanes 1–4 and ST in **a**.

We have previously shown and observed that a variety of cells grown in culture appear to remain genotypically relatively stable (van Helden & Wiid, 1987; van Helden *et al.*, 1988, and unpublished observations). This was also observed in fingerprints obtained from this line over a period of 6 months in continuous culture.

The BM 1604 cell line has an epithelial morphology, with loosely attached cells becoming more abundant with confluency. This appears to be characteristic of prostate cell lines (Kaighn *et al.*, 1979). The cells demonstrate staining of cytokeratins, similar to that in PPC-1 cells (Brothman *et al.*, 1989), and have no or low amounts of detectable PSA, as has been reported in PC-3 and DU-145 prostate cell lines, unlike the LNCaP cell line (Skowronski *et al.*, 1993). The number of prostate cell lines available for study has become even more limited since PPC-1 was recently reported to be a clonal derivative of PC-3 (Chen, 1993).

We conclude that BM 1604 is a new, unique prostate-derived cell line established using a new method, which may join the ranks of the few prostate lines available for molecular and cellular analyses.

The authors would like to thank R. Schneider for help with the cytogenetic aspects of the work, G.M. Hon for helping with the later culturing of the cells and The Cancer Association of South Africa for financial support.

**References**

ALI, S., MILLER, C.R. & EPPLEN, J.T. (1987). DNA fingerprinting by oligonucleotide probes specific for simple repeats. *Hum. Genet.*, **74**, 239-243.

ARMOUR, J.A.L., PATEL, I., THEIN, S.L., FEY, M.F. & JEFFREYS, A.J. (1989). Analysis of somatic mutations at human minisatellite loci in tumors and cell lines. *Genomics*, **4**, 328-334.

BETTINK, S., WULLICH, B., CHRISTMANN, A., ZIVERGEL, T., ZANG, K.-D. & UNTEREGGER, G. (1992). Genetic heterogeneity of prostatic carcinoma-derived cell lines as emphasized by DNA fingerprinting. *Electrophoresis*, **13**, 644-646.

BROTHMAN, A.R., LESHO, L.J., SOMERS, K.D., WRIGHT, G.L. & MERCHANT, D.J. (1989). Phenotypic and cytogenetic characterization of a cell line derived from primary prostatic carcinoma. *Int. J. Cancer*, **44**, 898-903.

CHEN, T.R. (1993). Chromosome identity of human prostate cancer cell lines, PC-3 and PPC-1. *Cytogenet. Cell Genet.*, **62**, 183-184.

DENIS, L. & MAHLER, C. (1990). Prostatic cancer (an overview). *Acta Oncol.*, **29**, 665-677.

EPPLEN, J.T. & ZISCHLER, H. (1990). *DNA Fingerprinting with Oligonucleotides*. Information sheet. Fresenius Diagnostik: Germany.

EPPLEN, J.T., KAMMERBAUER, C., STEIMLE, V., ZISCHLER, H., ALBERT, E., ANDREAS, A., HALA, K., NANDA, I., SCHMID, M., RIESS, O. & WEISING, K. (1989). Methodology and application of olignucleotide fingerprinting including characterization of individual hypervariable loci. In *Electrophoresis Forum '89: Proceedings of the International Meeting on Electrophoresis*, Technical University of Munich, October 23-25, 1989, Radola, B.J. (ed.) pp. 175-186. Bode-Verlag: Munchen.

FONG, C.J., SHERWOOD, E.R., SUTKOWSKY, D.M., ABU-JAWDEH, E.M., YAKOO, H., BAUER, K.D., KOZLOWSKI, J.M. & LEE, C. (1991). Reconstituted basement-membrane promotes morphological and functional differentiation of primary human prostatic epithelial cells. *Prostate*, **19**, 221-235.

GOLOMBICK, T. & BEZWODA, W.R. (1991). In vitro maintenance of a new ovarian-cancer cell-line in protein-free media – a potential model for autonomous growth and tumor progression. *Eur. J. Cell. Bio.*, **56**, 459-463.

KAIGHN, M.E., NARAYAN, K.S., OHNUKI, Y., LECHNER, J.F. & JONES, L.W. (1979). Establishment and characterization of a human prostatic carcinoma cell line (PC-3). *Invest. Urol.*, **17**, 16-23.

KAWAMOTO, T., SATO, J.D., LEE, A., MCCLURE, P.B. & SATO, G.H. (1983). Development of a serum-free medium for growth of NS-1 mouse myeloma cells and its application to the isolation of NS-1 hybridomas. *Ann. Biochem.*, **130**, 445-453.

LAGODA, P.J.L., SEITZ, G., EPPLEN, J.T. & ISSINGER, O.G. (1989). Increased detectability of somatic changes in the DNA from human tumours after probing with synthetic and genome-derived hypervariable multilocus probes. *Hum. Genet.*, **84**, 35-40.

LONGMURE, J.L., KRAEMER, P.M., BROWN, N.C., HARDEKOPF, L.C. & DEAVEN, L.L. (1989). A new multi-locus DNA fingerprinting probe: pV47-2. *Nucleic Acids Res.*, **18**, 1658.

LYTTON, B. (1989). Demographic factors in benign prostatic hyperplasia. In *The Prostate*, Fitzpatrick, J.M. & Krane, R.J. (eds). Churchill Livingstone: Edinburgh.

MATHEW, C.G.P. (1984). The isolation of high molecular weight eukaryotic DNA. In *Methods in Molecular Biology*. Vol. 2. Walker, J.M. (ed.) pp. 31-34, Humana Press: Clifton, NJ.

NELSON-REES, W.A., DANIELS, D.W. & FLANDERMEYER, R.R. (1981). Cross-contamination of cells in culture. *Science*, **212**, 446-452.

PIERRE, R.V. & HOAGLAND, H.C. (1972). Age-associated aneuploidy: loss of Y chromosome from human bone marrow cells with aging. *Cancer*, **30**, 889-894.

SKOWRONSKI, R.J., PEEHL, D.M. & FELDMAN, D. (1993). Vitamin D and prostate cancer: 1,25 dihydroxyvitamin $D_3$ receptors and actions in human prostate cancer cell lines. *Endocrinology*, **132**, 1952-1960.

SOUTHERN, E.M. (1975). Detection of specific sequences among DNA fragments separated by gel electrophoresis. *J. Mol. Biol.*, **98**, 503-518.

VAN HELDEN, P.D. & WIID, I.J.F. (1987). Application of DNA fingerprinting to the investigation of cell-line genotype. *SA J. Science*, **83**, 244.

VAN HELDEN, P.D., WIID, I.J.F., ALBRECHT, C.F., THERON, E., THORNLEY, A. & HOAL-VAN HELDEN, E.G. (1988). Cross-contamination of human esophageal squamous carcinoma cell lines detected by DNA-fingerprint analysis. *Cancer Res.*, **48**, 5660-5662.

VASSART, G., GEORGES, M., MONSIEUR, R., BROCAS, H., LEQUARRE, A.S. & CHRISTOPHE, D. (1987). A sequence in M13 phage detects hypervariable minisatellites in human and animal DNA. *Science*, **235**, 683-684.

WHITE, J.J., NEUWIRTH, H., MILLER, C.D. & SCHNEIDER, E.L. (1990). DNA alternations in prostatic adenocarcinoma and benign prostatic hyperplasia: detection by DNA fingerprint analyses. *Mutat. Res.*, **237**, 37-43.